# *Klebsiella* Species Associated with Bovine Mastitis in Newfoundland

**DOI:** 10.1371/journal.pone.0106518

**Published:** 2014-09-02

**Authors:** Milka P. Podder, Laura Rogers, Peter K. Daley, Greg P. Keefe, Hugh G. Whitney, Kapil Tahlan

**Affiliations:** 1 Department of Biology, Memorial University of Newfoundland, St. John’s, NL, Canada; 2 Animal Health Division, Newfoundland and Labrador Department of Natural Resources, St. John’s, NL, Canada; 3 Faculty of Medicine, Memorial University of Newfoundland, St. John’s, NL, Canada; 4 Department of Health Management, Atlantic Veterinary College, University of Prince Edward Island, Charlottetown, PEI, Canada; Auburn University, United States of America

## Abstract

*Klebsiella* spp. is a common cause of bovine mastitis, but information regarding its molecular epidemiology is lacking from many parts of the world. On using mass spectrometry and partial sequencing of the *rpo*B gene, it was found that over a one year study, *K. variicola* and *Enterobacter cloacae* were misidentified as *K. pneumoniae* in a small number of clinical mastitis (CM) cases from Newfoundland. Results suggest that the currently used standard biochemical/phenotypic tests lack the sensitivity required to accurately discriminate among the three mentioned Gram negative bacteria. In addition, a single strain of *K. variicola* was associated with CM from one farm in the study as demonstrated by Random Amplified Polymorphic DNA (RAPD) PCR. To the best of our knowledge, *K. variicola*, which is normally found in the environment, has not been isolated previously from milk obtained from cows with CM. Therefore, it is possible that *K. variicola* was not detected in milk samples in the past due to the inability of standard tests to discriminate it from other *Klebsiella* species.

## Introduction

*Klebsiella pneumoniae* is an important opportunistic human pathogen predominantly affecting immunocompromised or elderly human patients. Recently a hypervirulent *K. pneumoniae* strain was reported to be capable of causing fatal infections in healthy individuals [1]. Drug resistant forms of *K. pneumoniae*, especially those resistant to the β-lactam family of antibiotics are also a cause for concern due to the limited therapeutic options available for the treatment of such infections and the ability of these strains to rapidly spread and transfer the resistance phenotype [2]–[4]. In the dairy industry, *K. pneumoniae* is one of the known causes of primarily environment derived *Klebsiella* mastitis and has been the subject of numerous studies [3], [5]–[7]. Clinical mastitis (CM) is classified as the condition where an animal displays the physical symptoms of mastitis [8] and milk production and quality is also affected [9]. Whilst most studies show limited impact of treatment, Schukken *et al.* [10] reported a significant increase in bacteriological cure after the use of antimicrobials for treating non-severe cases of *Klebsiella* associated CM. Mastitis adversely affects milk production and generally cows do not regain full production levels post recovery [11], leading to considerable economic losses. It has also been reported that the amount of decrease in milk production depends on the specific pathogen causing the infection and that Gram negative bacteria are responsible for greater reduction than Gram positive bacteria and other non-bacterial organisms [11]–[14]. Although, not routinely performed for diagnostic purposes, further characterization of bacterial isolates from infected animals helps to better identify the sources of the infection and to determine if herd infections are primarily clonal or polyclonal in nature [3], [5], [7]. These data allow clinicians to understand the nature of transmission within herds and to implement targeted prevention strategies. Therefore, there is value in identifying the causative agent for quick action to prevent losses and also for surveillance purposes. In the current study, we used multiple methods to identify and analyze Gram negative coliforms associated with bovine CM from Newfoundland, Canada. Our results suggest that the currently used standard biochemical laboratory identification techniques were not sensitive enough to accurately identify the etiological agent in certain cases. In addition, we found that all CM cases from one farm were associated with *K. variicola*, which had been misidentified as *K. pneumoniae* using routine testing procedures.

## Materials and Methods

### Ethics statement

This study was carried out in cooperation with dairy farmers in the province and formalized by an agreement between their representative organization, the Dairy Farmers of Newfoundland and Labrador (NL), and the Chief Veterinary Officer for the Province of NL (HGW). Endangered species were not involved in the study and all samples were collected from the island of Newfoundland. The report is not intended to be a field study; instead it describes the application and accuracy of laboratory and molecular tests for the identification of coliform bacteria associated with bovine mastitis. Therefore, coordinates and details regarding the geographical origins of the samples are not included. Approval from an Institutional Animal Care and Use Committee (IACUC), or equivalent animal ethics committee, was not obtained as the samples used in the current study were obtained from routine veterinary diagnostic submissions unrelated to this research. The report is focused on the microbiological analysis following the isolation of bacteria from the milk samples, and did not directly involve any animals.

### Standard phenotypic/biochemical testing for pathogen identification

Milk samples from animals with symptoms of CM were collected from 11 farms by the Animal Health Division, Department of Natural Resources, Government of NL between October, 2011 and October, 2012. Sample collection from dairy cattle was done through the expression of milk from the teats into a sterile collection container for submission to the diagnostic laboratory. For initial confirmation of CM status of the animal, milk samples originating from infected quarters of the udder were subjected to the California mastitis test (CMT, Dairy Research Products Co., Ontario, Canada). For isolation and phenotypic identification of CM associated bacteria, milk samples were streaked on tryptic soy agar with 5% sheep blood, MacConkey 3 agar with Crystal Violet and urea agar (Oxoid Ltd., Ontario, Canada) as recommended (National Mastitis Council, 1999). To further characterize the isolates and to distinguish between members of Enterobacteriaceae, the IMViC test was performed using tryptic soy broth (I, Oxoid Ltd., Ontario, Canada), methyl red (M), Voges-Proskauer (V) test (Becton Dickinson, Sparks, Md.) and citrate test (C, Becton Dickinson, Sparks, Md.). The M test differentiates *Klebsiella* spp. from *Enterobacter cloacae* and the I test differentiates between *K. pneumoniae* and *K. oxytoca*. Atypical results were reanalyzed using the API 20E identification kit (bioMérieux Canada, Inc.). Later on, the isolates were submitted to the Public Health Laboratory of the Government of NL (PHL-NL, St. John’s, NL, Canada) for classification using the Bruker MALDI-TOF (Matrix-Assisted Laser Desorption/Ionization-Time Of Flight) Biotyper System (Bruker Corp. USA). Certain isolates were also tested for their ability to metabolize adonitol using the API 50 CHE kit according to the manufacturer’s instructions (bioMérieux Canada, Inc.).

### Antibiotic susceptibility testing

All isolates were subjected to routine drug susceptibility testing using cephalothin, ceftiofur, streptomycin, tetracycline and trimethoprim sulfamethoxazole (TMP-sulfa) using the Kirby Bauer Disc Diffusion method. Minimum inhibitory concentrations were also determined for cephalothin, ceftiofur and tetracycline using the Sensititer microdilution system (Trek Diagnostic Systems Inc.). All analysis was conducted according to the Clinical and Laboratory Standards Institute (CLSI) standards [15].

### Genotyping of isolates

The isolates were successfully grown in nutrient broth (Becton Dickinson, Sparks, Md.) for molecular typing. Chromosomal DNA was extracted from the 45 isolates and used for direct amplification and sequencing of the *rpo*B gene as described earlier [5] using the Thermo Scientific Phusion High-Fidelity PCR Kit. PCR products were subjected to DNA sequencing at the Centre for Applied Genomics, University of Toronto, Canada and the nucleotide sequences obtained were used to search the public database (http://blast.ncbi.nlm.nih.gov/Blast.cgi). Forty five isolates from the current study were used for strain typing by Random Amplified Polymorphic DNA (RAPD) PCR as described earlier [5], [16]. The primer pair was designed to analyze different species of *Klebsiella* [17]. Images of DNA banding patterns obtained after agarose gel electrophoresis were analyzed using the PyElph software [18] to prepare dendrograms using the neighbor joining method. Reproducibility of banding patterns for 45 isolates was also evaluated.

## Results and Discussion

In the current study, *Klebsiella* spp. were detected in 61 milk samples that were routinely collected from cows with symptoms of CM from 11 farms in Newfoundland over a one year period. From these original 61 isolates that were identified as *K. pneumoniae* based on standard biochemical and phenotypical tests, only 45 could be re-cultured for further examination. Subsequent analysis of all the isolates by MALDI-TOF Biotyper mass spectrometry reclassified two of the 45 isolates as *Enterobacter cloacae* and the remaining 43 as *K. pneumoniae* (Table 1). To determine/confirm genus and species identity, partial sequencing of the *rpo*B gene was carried out as it has been shown to be a good candidate to discriminate between coliforms associated with CM in previous studies [5]. The DNA sequences obtained were aligned after trimming and were used to build a dendrogram (Figure S1). In this third round of analysis, all CM associated samples from a single farm (farm 9) were identified as *K. variicola* and not *K. pneumoniae*. In addition, two isolates from separate farms were confirmed to be *E. cloacae*, as suggested previously by MALDI-TOF analysis (Table 1). Therefore, *K. pneumoniae*, *K. variicola* and *E. cloacae* were identified to be associated with CM cases from 11 farms in the current study.

**Table 1:** Detailed genus and species level identification of 45 coliform isolates obtained from milk samples from 11 Newfoundland dairy farms using the different identification methods described in the current study.

Farm of origin and number of isolates^a^	Identification^b^
Farm 1 (26)	All *K. pneumoniae*
Farm 2 (1)	All *K. pneumoniae*
Farm 3 (5)^c^	4 *K. pneumoniae and* 1 *E. cloacae* d
Farm 4 (2)	All *K. pneumoniae*
Farm 5 (3)^c^	2 *K. pneumoniae and* 1 *E. cloacae* d
Farm 6 (2)	All *K. pneumoniae*
Farm 7 (1)	All *K. pneumoniae*
Farm 8 (1)	All *K. pneumoniae*
Farm 9 (2)^e^	All *K. variicola* f
Farm 10 (1)	All *K. pneumoniae*
Farm 11 (1)	All *K. pneumoniae*

a Number of pure culture isolates from CM cases from each farm are shown in parenthesis.

b Final identification using a combination of the methods (biochemical/phenotypical tests, MALDI-TOF and *rpo*B sequencing) are shown.

c Farms showed the presence of cephalothin-resistant *E. cloacae.*

d *E. cloacae* could be identified using both MALDI-TOF and *rpo*B sequencing, but not by standard biochemical/phenotypic methods.

e All isolates from this farm were identified as adonitol fermenting *K. variicola.*

f *K. variicola* could be only identified by *rpo*B sequencing, but not by standard biochemical/phenotypic methods or by MALDI-TOF.

Only *K. variicola* was isolated from cows with CM from farm 9, originating from the left hind quarter of the udders of two animals. One of the strains (KM-49) was re-isolated from the same quarter during a second sampling conducted after a one month period. *K. variicola* was originally described to be unable to metabolize/ferment adonitol [20]. When the two *K. variicola* isolates from the current study were used in carbohydrate fermentation assays, results demonstrated that both isolates could ferment adonitol (Table S1). Therefore, in the current study, *K. variicola* could only be identified based on *rpo*B sequencing, as all other methods, including the adonitol fermentation test failed to identify it.

**Figure 1:**
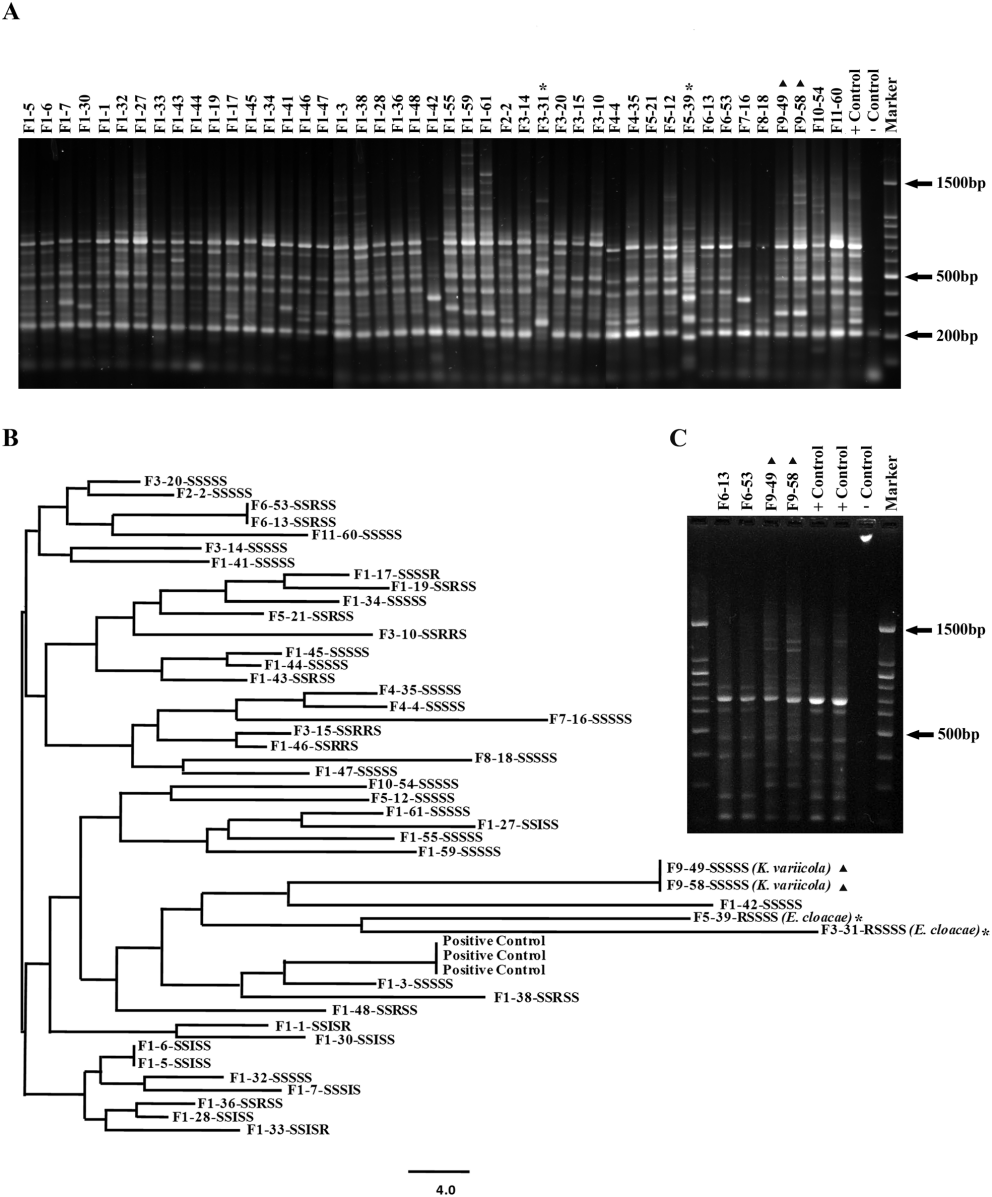
Molecular epidemiology of CM associated coliform bacteria isolated over a one year period from 11 dairy herds in Newfoundland. (**A**) Results from RAPD analysis employing 1.5% agarose gels to separate PCR products. Chromosomal DNA from a laboratory strain (*K. pneumoniae* ATCC 15380) was included as the positive control and sterile water functioned as negative control in the PCR assays. A 100 bp ladder (Cat No. DM001-R500, GeneDirex, USA) was used as the molecular weight marker (**B**) Dendrogram based on RAPD results showing relatedness between strains subjected to analysis in the current study. The dendrogram was generated by the neighbor joining method with the PyElph software. (**A, B and C**) The isolates were assigned labels based on the farm or origin (F1 to F11) followed by a number to identify the infected animal. The isolates determined to be *E. cloacae* (*) and *K. variicola* (^▴^) are also indicated. (**B**) The results from antibiotic susceptibility testing are also shown next to each isolate. S: sensitive, I: intermediate and R: resistant designations are assigned based on established break points. The S/I/R designations for each drug are in the following order: cephalothin, ceftiofur, streptomycin, tetracycline and trimethoprim sulfamethoxazole, respectively. The isolates identified as *K. variicola* and *E. cloacae* on further analysis are also indicated. (**C**) Re-analysis of strains by RAPD PCR to verify the accuracy of the assay and to confirm identities. PCR products generated independently for the second time using DNA from identical strains along with the positive control were reanalyzed, which confirmed results shown in **A**.

Many diverse *K. pneumoniae* strains are known to be present in dairy cattle feces and infections are normally linked to contaminated organic bedding material [16]. Genotyping techniques such as RAPD, Multilocus Sequence Typing (MLST) and Pulsed-field Gel Electrophoresis (PFGE) have been used in the past to successfully analyze *K. pneumoniae* strain diversity associated with CM [5], [21], [22]. Of these, RAPD has many advantages as it is fast, relatively inexpensive and technically less demanding as compared to the other methods of analysis and was therefore chosen for the current study. Limited strain clustering was observed between the *K. pneumoniae* strains subjected to analysis (Figure 1), which has also been reported in previous studies, suggesting an environmental source of the infection [16], [19], [21], with the exception of farm 6, where the two CM cases were associated with the same *K. pneumoniae* strain (Figure 1). Therefore, it is possible that there was either direct or indirect animal to animal transmission on farm 6 or that a single environmental strain infected the two animals independently. Chromosomal DNA from the laboratory *K. pneumoniae* ATCC 15380 strain, was used as a control for RAPD analysis on two occasions, giving identical profiles, showing that the assay used is reliable enough for strain typing and all results were reproducible (Figure 1C). Examination of the two *K. variicola* isolates from farm 9 demonstrated that they were identical, which could again suggest animal to animal transmission or that a single strain infected the two animals independently (Figure 1). Similar results were also reported in previous studies where RAPD demonstrated clear difference between *Raoultella* spp. and *K. pneumoniae* using the same pair of primers as used in the current study [5]. The RAPD assay was repeated to check for reproducibility and verify the DNA banding patterns between identical isolates (Figure 1C). In addition, the two *E. cloacae* strains identified in the current study from two different farms were not the same (Figure 1A and 1B).

All 45 isolates were also subjected to antimicrobial susceptibility testing using five drugs commonly used to treat Gram negative infections in veterinary medicine (cephalothin, ceftiofur, streptomycin, tetracycline and TMP-sulfa). CLSI guidelines were used for determining the breakpoint concentration for each antibiotic. Cephalothin resistance (a first-generation cephalosporin β-lactam) was only observed in the *E. cloacae* isolates. Combined results from these analyses are superimposed on the dendrogram shown in Figure 1B and are also included in Table S2. Varying profiles/degrees of resistance against streptomycin and tetracycline were observed for the *K. pneumoniae* isolates. In addition, three isolates from farm 1 (F1-1, F1-17 and F1-33) were resistant to TMP-sulfa and the two *K. variicola* isolates were sensitive to all the drugs tested (Figure 1B and Table S2).

The insular nature of Newfoundland, its location and maritime climate pose unique challenges and environments for the management of dairy herds. In the described study, bacteria were isolated from confirmed CM dairy animals from Newfoundland and were initially identified as *K. pneumoniae* using standard phenotypic laboratory testing protocols. MALDI-TOF finger printing further reclassified two isolates as *E. cloacae*, suggesting that this method is more sensitive than phenotypic biochemical analysis in discriminating between coliforms associated with CM [23]. MALDI-TOF has been used for proteomic analysis in several studies related to bovine mastitis [24], [25]. MALDI-TOF is a rapid, precise and cost-effective method for bacterial identification compared to conventional phenotypic/biochemical techniques [26], but its sensitivity is dependent on the database used for matching the obtained spectra. Finally, partial *rpo*B gene sequencing revealed the presence of *K. variicola* from one farm, which could not be discriminated using the two previous methods. Similar results were also observed in previous studies where *Klebsiella oxytoca*, *Klebsiella variicola* and *Raoultella planticola* were isolated from environmental samples associated with CM [19]. Therefore, it is possible that the prevalence of *K. variicola* associated with CM might be under-reported, as the results suggest that routinely used identification tests are not sensitive enough to discriminate it from *K. pneumoniae*.

*K. pneumoniae* is an opportunistic, environmental pathogen causing CM in dairy cattle [16] and it is very rare to see a dominant strain associated with a herd [5]. Farm 1, which had the highest incidence of CM over the period included in the study, displayed large amounts of variation in *K. pneumoniae* strains and only farm 6 had infections caused by a single strain (Figure 1). Results also showed that both cases from farm 9 were associated with a single strain of *K. variicola* (Figure 1) as it was the only bacterium cultured from the submitted milk samples (Table S2). The isolation of *K. variicola* from milk samples from cows with CM has not been reported previously as this organism is normally found in the environment [19]. In addition, *K. variicola* has also been previously isolated from plants and certain hospital settings [19], [20]. Other reports have questioned the adonitol negative fermentation test for its ability to discriminate between *K. variicola* and other coliforms [27], which was also demonstrated by our results. The pathogenic potential of *K. variicola* is not well understood and there has been some concern in the ability to accurately detect it in humans. In a recent report, an incorrectly diagnosed *K. variicola* strain was responsible for human patient mortality, even though antibiotics were administered to which the isolate was sensitive under laboratory conditions [28]. To the best of our knowledge, this is the first report with evidence that an isolate of *K. variicola* can cause CM in dairy cattle, as it is normally found in soil and feed [19], and not in milk from infected animals. The relevance of the finding that one adonitol positive strain of *K. variicola* was responsible for both CM cases from a single farm will be investigated in future studies.

## Supporting Information

Figure S1 **Phylogenetic tree derived from the** ***rpo*** **B partial gene sequences of all 45 isolates built using the neighbour joining method in the Molecular Evolutionary Genetic Analysis (MEGA) package (version 6.06).** Grouping of sequences of all the isolates were based on % confidence obtained by using a boot-strap value of 1000. The isolates were assigned labels based on the farm or origin (F1 to F11) followed by a number to identify the infected animal. The results of antibiotic susceptibility testing are also shown beside each isolate. S: sensitive, I: intermediate and R: resistant, based on Clinical and Laboratory Standards Institute (CLSI) interpretation. The S/I/R designations for each antibiotic are in the following order: cephalothin, ceftiofur, streptomycin, tetracycline and trimethoprim sulfamethoxazole, respectively. The isolates identified as *K. variicola*, *E. cloacae* and *K. pneumoniae* are also indicated.
(DOCX)

Table S1 **Results of tests using** ***K. variicola*** **isolates for their ability to metabolize select carbohydrates including adonitol, using the API 50 CHE kit after 24 hours of incubation (bioMérieux, Inc.).**
(DOCX)

Table S2 **Details regarding animal/farm ID, sample collection date, infected quarter sampled and California Mastitis Test (CMT) results.** Information regarding bacterial identification by various methods (culture/biochemical tests, *rpo*B sequencing and MALDI-TOF), and antibiotic susceptibility test results (by minimum inhibitory concentrations and Kirby Bauer disc diffusion method according to CLSI guideline) are also included. Other organisms that were present in the samples besides *K. pneumoniae* based on biochemical/phenotypic methods are also indicated.
(DOCX)
