# Supplementary material for: Klebsiella Species Associated with Bovine Mastitis in Newfoundland
Source: PLoS One. 2014 Sep 2;9(9):e106518. doi: 10.1371/journal.pone.0106518 (PMC4152263; doi:10.1371/journal.pone.0106518)

**Figure S1.** Phylogenetic tree derived from the *rpo*B partial gene sequences of all 45 isolates built using the neighbour joining method in the Molecular Evolutionary Genetic Analysis (MEGA) package (version 6.06). Grouping of sequences of all the isolates were based on % confidence obtained by using a boot-strap value of 1000. The isolates were assigned labels based on the farm or origin (F1 to F11) followed by a number to identify the infected animal. The results of antibiotic susceptibility testing are also shown beside each isolate. S: sensitive, I: intermediate and R: resistant, based on Clinical and Laboratory Standards Institute (CLSI) interpretation. The S/I/R designations for each antibiotic are in the following order: cephalothin, ceftiofur, streptomycin, tetracycline and trimethoprim sulfamethoxazole, respectively. The isolates identified as *K. variicola*, *E. cloacae* and *K. pneumoniae* are also indicated.


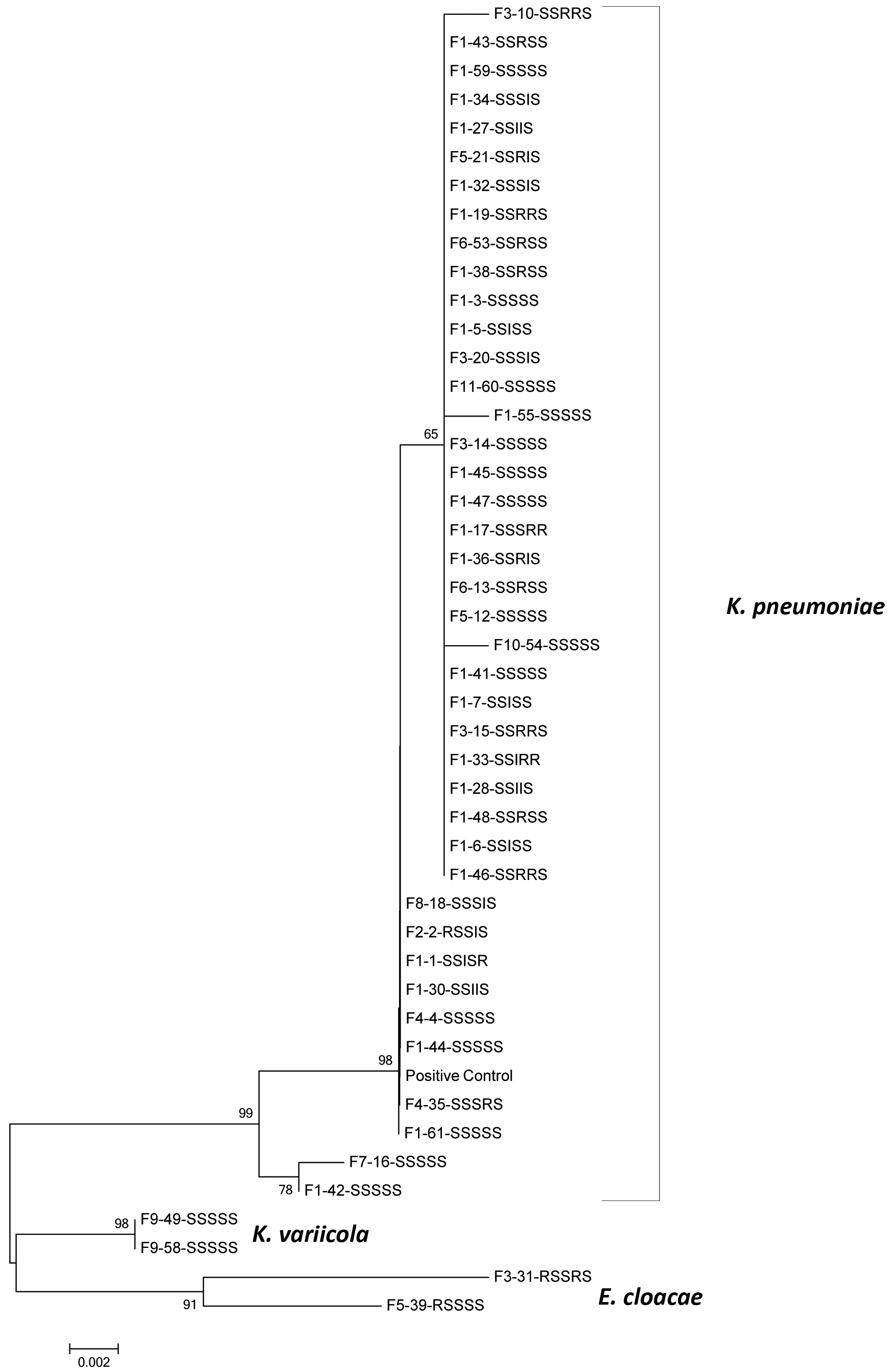

Supplement: Figure S1 — Phylogenetic tree derived from the rpo B partial gene sequences of all 45 isolates built using the neighbour joining method in the Molecular Evolutionary Genetic Analysis (MEGA) package (version 6.06). Grouping of sequences of all the isolates were based on % confidence obtained by using a boot-strap value of 1000. The isolates were assigned labels based on the farm or origin (F1 to F11) followed by a number to identify the infected animal. The results of antibiotic susceptibility testing are also shown beside each isolate. S: sensitive, I: intermediate and R: resistant, based on Clinical and Laboratory Standards Institute (CLSI) interpretation. The S/I/R designations for each antibiotic are in the following order: cephalothin, ceftiofur, streptomycin, tetracycline and trimethoprim sulfamethoxazole, respectively. The isolates identified as K. variicola, E. cloacae and K. pneumoniae are also indicated. (DOCX) [file pone.0106518.s001.docx]
