# Supplementary material for: Klebsiella Species Associated with Bovine Mastitis in Newfoundland
Source: PLoS One. 2014 Sep 2;9(9):e106518. doi: 10.1371/journal.pone.0106518 (PMC4152263; doi:10.1371/journal.pone.0106518)
Supplement: Table S1 — Results of tests using K. variicola isolates for their ability to metabolize select carbohydrates including adonitol, using the API 50 CHE kit after 24 hours of incubation (bioMérieux, Inc.). (DOCX) [file pone.0106518.s002.docx]

**Table S1.** Results of tests using *K. variicola* isolates for their ability to metabolize select carbohydrates including adonitol, using the API 50 CHE kit after 24 hours of incubation (bioMérieux, Inc.).

| **Sample** | | ***K. pneumoniae* (cont) ^a^** | | | **F9-49** | | | **F9-58** | | |
| --- | --- | --- | --- | --- | --- | --- | --- | --- | --- | --- |
| **No.** | **Test** | **Color ^b^** | **Result ^c^** | **Bubbles ^d^** | **Color ^b^** | **Result ^c^** | **Bubbles ^d^** | **Color ^b^** | **Result ^c^** | **Bubbles ^d^** |
| 0 | 0 | R | ⁻ | A | R | ⁻ | A | R | ⁻ | A |
| 1 | GLY | Y | ⁺ | P | Y | ⁺ | P | Y | ⁺ | A |
| 2 | ERY | R | ⁻ | A | R | ⁻ | A | R | ⁻ | A |
| 3 | DARA | R | ⁻ | A | R | ⁻ | A | R | ⁻ | A |
| 4 | LARA | Y | ⁺ | P | Y | ⁺ | P | Y | ⁺ | P |
| 5 | RIB | Y | ⁺ | A | Y | ⁺ | P | Y | ⁺ | A |
| 6 | DXYL | Y | ⁺ | P | Y | ⁺ | P | Y | ⁺ | P |
| 7 | LXYL | R | ⁻ | A | R | ⁻ | A | R | ⁻ | A |
| **8** | **ADO ^e^** | **Y** | **⁺** | **P** | **Y** | **⁺** | **A** | **Y** | **⁺** | **A** |
| 9 | MDX | R | ⁻ | A | R | ⁻ | A | R | ⁻ | A |
| 10 | GAL | Y | ⁺ | P | Y | ⁺ | P | Y | ⁺ | P |
| 11 | GLU | Y | ⁺ | P | Y | ⁺ | P | Y | ⁺ | P |
| 12 | FRU | Y | ⁺ | P | Y | ⁺ | P | Y | ⁺ | P |
| 13 | MNE | Y | ⁺ | P | Y | ⁺ | P | Y | ⁺ | P |
| 14 | SBE | Y | ⁺ | P | Y | ⁺ | P | Y | ⁺ | P |
| 15 | RHA | Y | ⁺ | A | Y | ⁺ | P | Y | ⁺ | A |
| 16 | DUL | R | ⁻ | A | Y | ⁺ | P | Y | ⁺ | P |
| 17 | INO | Y | ⁺ | P | Y | ⁺ | A | Y | ⁺ | P |
| 18 | MAN | Y | ⁺ | P | Y | ⁺ | P | Y | ⁺ | P |
| 19 | SOR | Y | ⁺ | P | Y | ⁺ | A | Y | ⁺ | P |
| 20 | MDM | R | ⁻ | A | R | ⁻ | A | R | ⁻ | A |
| 21 | MDG | Y | ⁺ | A | Y | ⁺ | P | Y | ⁺ | A |
| 22 | NAG | O | ⁺ | P | O | ⁺ | P | O | ⁺ | A |
| 23 | AMY | R | ⁻ | A | R | ⁻ | A | R | ⁻ | A |
| 24 | ARB | Y | ⁺ | P | Y | ⁺ | P | Y | ⁺ | P |
| 25 | ESC | B | ⁻ | A | B | ⁻ | P | B | ⁻ | A |
| 26 | SAL | Y | ⁺ | P | Y | ⁺ | P | Y | ⁺ | P |
| 27 | CEL | Y | ⁺ | P | Y | ⁺ | P | Y | ⁺ | P |
| 28 | MAL | Y | ⁺ | P | Y | ⁺ | P | Y | ⁺ | P |
| 29 | LAC | Y | ⁺ | P | Y | ⁺ | P | Y | ⁺ | P |
| 30 | MEL | Y | ⁺ | P | Y | ⁺ | P | Y | ⁺ | P |
| 31 | SAC | Y | ⁺ | P | Y | ⁺ | P | Y | ⁺ | A |
| 32 | TRE | Y | ⁺ | P | Y | ⁺ | P | Y | ⁺ | P |
| 33 | INU | R | ⁻ | A | R | ⁻ | A | R | ⁻ | A |
| 34 | MLZ | R | ⁻ | A | R | ⁻ | A | R | ⁻ | A |
| 35 | RAF | Y | ⁺ | P | Y | ⁺ | P | Y | ⁺ | P |
| 36 | AMD | Y | ⁺ | P | R | ⁻ | A | R/O | V | A |
| 37 | GLYG | R | ⁻ | A | R | ⁻ | A | R | ⁻ | A |
| 38 | XLT | R | ⁻ | A | R | ⁻ | A | R | ⁻ | A |
| 39 | GEN | Y | ⁺ | P | Y | ⁺ | P | Y | ⁺ | A |
| 40 | TUR | R | ⁻ | A | O | ⁺ | A | R | ⁻ | A |
| 41 | LYX | R | ⁻ | A | O | ⁺ | A | R | ⁻ | A |
| 42 | TAG | Y | ⁺ | P | Y | ⁺ | P | Y | ⁺ | P |
| 43 | DFUC | R | ⁻ | A | R | ⁻ | A | R | ⁻ | A |
| 44 | LFUC | Y | ⁺ | P | Y | ⁺ | P | Y | ⁺ | A |
| 45 | DARL | Y | ⁺ | P | Y | ⁺ | P | Y | ⁺ | P |
| 46 | LARL | R | ⁻ | A | R | ⁻ | A | R | ⁻ | A |
| 47 | GNT | O | ⁺ | P | O | ⁺ | P | R | ⁻ | P |
| 48 | 2KG | O | ⁺ | A | O | ⁺ | P | R/O | V | P |
| 49 | 5KG | R | ⁻ | A | Y/O | V | P | O | ⁺ | P |

**^a^** Laboratory strain *K. pneumoniae* ATCC 15380 was used in the analysis as a control

**^b^** Colour: R = red and Y = yellow

**^c^** Result: + = positive, - = negative and v = variable

**^d^** Bubbles: A = absent and P = present

**^e^** ADO = adonitol

All interpretation are based on the manufacturers recommendations
