# Supplementary material for: Klebsiella Species Associated with Bovine Mastitis in Newfoundland
Source: PLoS One. 2014 Sep 2;9(9):e106518. doi: 10.1371/journal.pone.0106518 (PMC4152263; doi:10.1371/journal.pone.0106518)
Supplement: Table S2 — Details regarding animal/farm ID, sample collection date, infected quarter sampled and California Mastitis Test (CMT) results. Information regarding bacterial identification by various methods (culture/biochemical tests, rpoB sequencing and MALDI-TOF), and antibiotic susceptibility test results (by minimum inhibitory concentrations and Kirby Bauer disc diffusion method according to CLSI guideline) are also included. Other organisms that were present in the samples besides K. pneumoniae based on biochemical/phenotypic methods are also indicated. (DOCX) [file pone.0106518.s003.docx]

**Table** **S2.** Details regarding animal/farm ID, sample collection date, infected quarter sampled and California Mastitis Test (CMT) results. Information regarding bacterial identification by various methods (culture/biochemical tests, *rpo*B sequencing and MALDI-TOF), and antibiotic susceptibility test results (by minimum inhibitory concentrations and Kirby Bauer disc diffusion method according to CLSI guideline) are also included. Other organisms that were present in the samples besides *K. pneumoniae* based on biochemical/phenotypic methods are also indicated.

| **Lab ID** | **Farm/**  **Animal ID** | **Collection Date** | **Qu^a^** | **CMT^b^** | **Growth on MacConkey 3 agar with crystal violet** | **Identification of isolates by various methods^c^** | | | **Antibiotic susceptibility**  **Test result^e^** | | | | | **Other organisms present (if any)** |
| --- | --- | --- | --- | --- | --- | --- | --- | --- | --- | --- | --- | --- | --- | --- |
|  |  |  |  |  |  | ***rpo*B** | **Phenotype** | **MALDI-TOF (Score value)^d^** | **CEF^f^** | **CF^g^** | **S^h^** | **TE^i^** | **TS^j^** |  |
| 1 | 001-007 | 6-Oct-11 | LH | 3+ | Heavy | + | + | + (2.397) | S | S | I | S | R | *Streptococcus uberis* **^k^** |
| 2 | 002-001 | 6-Oct-11 | RH | N/A | Very Light | + | + | + (2.526) | S | S | S | S | S | None |
| 3 | 001-022 | 10-Nov-11 | LH | 3+ | Light | + | + | + (2.435) | S | S | S | S | S | Mixed **^k^** |
| 4 | 004-001 | 12-Nov-11 | RF | 2+ | Very Light | + | + | + (2.43) | S | S | S | S | S | None |
| 5 | 001-001 | 21-Nov-11 | NG | 3+ | Heavy | + | + | + (2.18) | S | S | I | S | S | None |
| 6 | 001-002 | 17-Nov-11 | LF | 3+ | Heavy | + | + | + (2.234) | S | S | I | S | S | None |
| 7 | 001-004 | 15-Nov-11 | RF | N/A | Light | + | + | + (2.456) | S | S | I | S | S | None |
| 10 | 003-006 | 13-Dec-11 | LH | 3+ | Scanty | + | + | + (2.382) | S | S | R | R | S | None |
| 12 | 005-003 | 3-Jan-12 | RH | 3+ | Heavy | + | + | + (2.488) | S | S | S | S | S | None |
| 13 | 006-004 | 28-Dec-11 | RF | 2+ | Heavy | + | + | + (2.446) | S | S | R | S | S | None |
| 14 | 003-002 | 6-Jan-12 | RH | N/A | Heavy | + | + | + (2.516) | S | S | S | S | S | None |
| 15 | 003-005 | 6-Jan-12 | RH | N/A | Heavy | + | + | + (2.504) | S | S | R | R | S | *E. coli* **^k^** |
| 16 | 007-001 | 17-Jan-12 | RH | N/A | Very scanty | + | + | + (2.334) | S | S | S | S | S | None |
| 17 | 001-015 | 16-Feb-12 | RH | N/A | Heavy | + | + | + (2.473) | S | S | S | S | R | None |
| 18 | 008-001 | 28-Feb-12 | NG | 2+ | Light | + | + | + (2.236) | S | S | S | S | S | Mixed **^k^** |
| 19 | 001-014 | 25-Feb-12 | RH | N/A | Heavy | + | + | + (2.466) | S | S | R | S | S | None |
| 20 | 003-004 | 7-Mar-12 | 1Q | 3+ | Moderate | + | + | + (2.425) | S | S | S | S | S | None |
| 21 | 005-002 | 9-Mar-12 | LF | 2+ | Very Light | + | + | + (2.494) | S | S | R | S | S | None |
| 27 | 001-009 | 5-Apr-12 | RF | N/A | Moderate | + | + | + (2.481) | S | S | I | S | S | None |
| 28 | 001-024 | 6-Apr-12 | LF | N/A | Moderate | + | + | + (2.485) | S | S | I | S | S | None |
| 30 | 001-006 | 10-May-12 | RF | N/A | Heavy | + | + | + (2.426) | S | S | I | S | S | None |
| 31 | 003-003 | 29-May-12 | LH | 3+ | Heavy | - | + | - (2.027) | R | S | S | S | S | None |
| 32 | 001-008 | 16-May-12 | RH | N/A | Very light | + | + | + (2.39) | S | S | S | S | S | None |
| 33 | 001-010 | 20-May-12 | LH | N/A | Heavy | + | + | + (2.477) | S | S | I | S | R | None |
| 34 | 001-017 | 22-May-12 | RF | N/A | Very Light | + | + | + (2.507) | S | S | S | S | S | None |
| 35 | 004-002 | 21-Jun-12 | LH | 3+ | Light | + | + | + (2.448) | S | S | S | S | S | Mixed **^k^** |
| 36 | 001-025 | 21-Jun-12 | RH | N/A | Scanty | + | + | + (2.481) | S | S | R | S | S | None |
| 38 | 001-023 | 23-Jul-12 | RF | N/A | Very scanty | + | + | + (2.443) | S | S | R | S | S | None |
| 39 | 005-004 | 2-Aug-12 | NG | 3+ | Moderate | - | + | - (2.348) | R | S | S | S | S | coagulase negative *Staphlococcus* **^k^** |
| 41 | 001-018 | 14-Aug-12 | RF | N/A | Very Light | + | + | + (2.483) | S | S | S | S | S | None |
| 42 | 001-027 | 13-Sep-12 | RF | N/A | Heavy | + | + | + (2.396) | S | S | S | S | S | None |
| 43 | 001-012 | 13-Sep-12 | RF | N/A | Scanty | + | + | + (2.487) | S | S | R | S | S | None |
| 44 | 001-013 | 13-Sep-12 | LH | N/A | Scanty | + | + | + (2.481) | S | S | S | S | S | None |
| 45 | 001-016 | 13-Sep-12 | LH | N/A | Moderate | + | + | + (2.423) | S | S | S | S | S | *E. coli* **^k^** |
| 46 | 001-019 | 13-Sep-12 | RF | N/A | Heavy | + | + | + (2.358) | S | S | R | R | S | Mixed **^k^** |
| 47 | 001-020 | 13-Sep-12 | RF | N/A | Scanty | + | + | + (2.499) | S | S | S | S | S | None |
| 48 | 001-026 | 13-Sep-12 | RH | N/A | Scanty | + | + | + (2.475) | S | S | R | S | S | None |
| 49 | 009-001 | 20-Sep-12 | LH | 3+ | Scanty | - | + | + (2.385) | S | S | S | S | S | None |
| 53 | 006-006 | 13-Aug-12 | LH | N/A | Scanty | + | + | + (2.508) | S | S | R | S | S | None |
| 54 | 010-001 | 3-Oct-12 | NG | 3+ | Light | + | + | + (2.422) | S | S | S | S | S | None |
| 55 | 001-029 | 1-Oct-12 | LF | N/A | Moderate | + | + | + (2.446) | S | S | S | S | S | None |
| 58 | 009-004 | 27-Oct-12 | LH | 2+ | Moderate | - | + | + (2.372) | S | S | S | S | S | None |
| 59 | 001-030 | 10-Oct-12 | LF | N/A | Light | + | + | + (2.521) | S | S | S | S | S | None |
| 60 | 011-001 | 26-Oct-12 | LH | 3+ | Scanty | + | + | + (2.449) | S | S | S | S | S | None |
| 61 | 001-031 | 19-Oct-12 | RF | N/A | Heavy | + | + | + (2.48) | S | S | S | S | S | None |

**^a^** Infected quarter of the udder sampled: RF = right forward, LF = Left forward, RH = right hind, LH = Left hind, 1Q = one quarter and NG = not given

**^b^** California Mastitis Test result: N/A = not applicable (as milk samples were frozen upon arrival), 2+/ 3+ = positive result. The reaction of CMT is scored on a scale of 0 (mixture liquid, no precipitate) to 3 (almost-solid gel forms) where 2+ means distinct gel formation and 3+ is strong gel formation that tends to adhere to paddle

**^c^** Identification of isolates by various methods: + = positive for *K. pneumoniae* and - = negative for *K. pneumoniae*

**^d^** MALDI-TOF (Range of score value): 2.3-3.00 = high probable species identification, 2.00-2.99 = secure genus identification, probable species identification

**^e^** Antibiotic susceptibility Test result: S = sensitive, I = intermediate and R = resistant

**^f^** CEF: ceftiofur

**^g^** CF: cephalothin

**^h^** S: streptomycin

**^i^** TE: tetracycline

**^j^** TS: trimethoprim sulfamethoxazole

**^k^** In some cases other organisms were also detected in the milk samples, which could not be identified in certain instances
